# Bioinspired Wet Adhesive Proanthocyanidins Microneedles for Ocular Wound Healing

**DOI:** 10.34133/research.0485

**Published:** 2024-09-24

**Authors:** Bin Kong, Rui Liu, Tiantian Kong, Yuanjin Zhao

**Affiliations:** ^1^ School of Biomedical Engineering, Shenzhen University Medical School, Shenzhen University, Shenzhen 518060, China.; ^2^ Department of Rheumatology and Immunology, Nanjing Drum Tower Hospital, School of Biological Science and Medical Engineering, Southeast University, Nanjing 210096, China.; ^3^ Department of Urology, The First Affiliated Hospital of Shenzhen University, Shenzhen Second People’s Hospital, Shenzhen 518000, China.; ^4^ Shenzhen Research Institute, Southeast University, Shenzhen 518071, China

## Abstract

Microneedles have shown considerable potential in treating ocular diseases, yet enhancing their architecture and functionality to improve therapeutic efficacy poses marked challenges. Here, inspired by the antioxidant strategy of blueberries and the wet adhesive mechanism of clingfish, we construct hierarchical and multifunctional microneedles. These microneedles possess both wet adhesive and antioxidant properties, making them highly effective for ocular wound healing. Constructed using polyacrylic acid-*N*-hydroxysuccinimide-based hydrogel with hexagonal structures, these generated microneedles ensure strong adhesion in wet environments. Furthermore, by incorporating proanthocyanidins (pAc) into the tips, the microneedle is imparted with excellent competence to scavenge reactive oxygen species (ROS). In the rat model of ocular alkali burns, the designed microneedle not only exhibited robust adhesion and desirable antioxidant properties in the moist ocular environment but also facilitated sustained drug release and effective treatment. These results suggest that our bioinspired microneedles with multifunctional properties offer substantial advancement over conventional approaches, positioning them as promising candidates for versatile wound healing applications.

## Introduction

Ocular chemical injuries, particularly alkali burns, represent a critical emergency in ophthalmology, necessitating immediate assessment and treatment [1,2]. Failure to effectively address these injuries may result in severe complications, including dry eye, inflammation, corneal opacity, neovascularization, and even damage to retinal and optic nerve [3]. Notably, oxidative stress plays a crucial role in the progression of alkali burns [4]. Inflammation can cause the production of large amounts of reactive oxygen species (ROS), leading to a virulent cycle of inflammation after alkali burns [5]. Therefore, sustained delivery of a highly efficient antioxidant agent to the ocular surface becomes an essential strategy for treating ocular alkali burns. Traditionally, a protective membrane embedded with drugs has been applied to the damaged area. However, the efficacy of this approach is challenged by the diluting effect of tears, complicating effective drug delivery to ocular tissues [6,7]. Microneedles (MNs) have emerged as an innovative solution, as they are extensively employed for penetrating the epidermis to achieve painless, noninvasive, and effective transdermal drug delivery [8–13]. However, the efficacy of most MNs is often compromised in highly mobile or moist tissues, where they may not adhere well and are prone to detachment [14–17]. Thus, a novel MN system with ROS scavenging ability is highly desirable for treating ocular alkali burns.

**Fig. 1.**
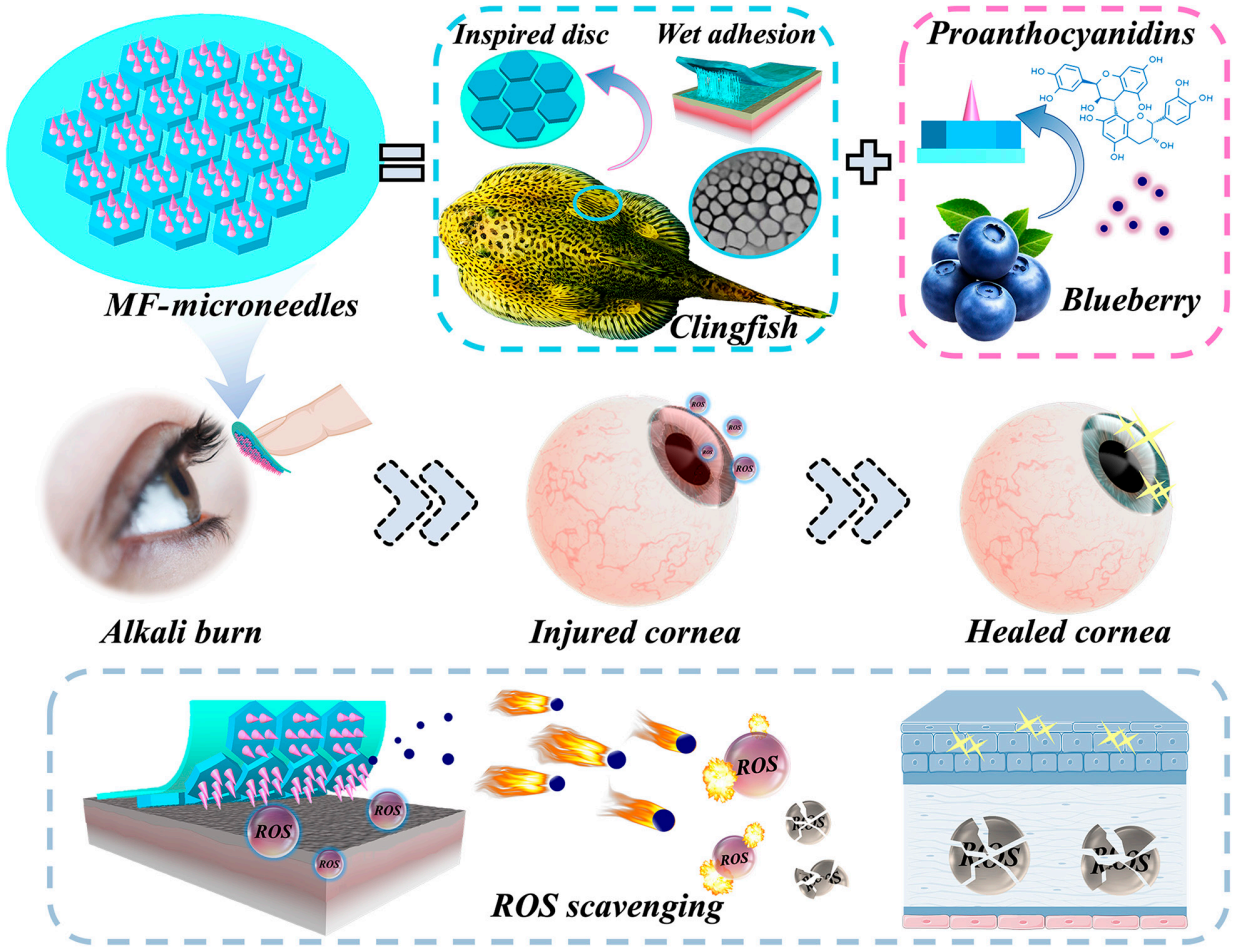
The scheme exhibits the components of the MF-microneedles and their applications in the treatment of alkali burns.

In this paper, inspired by the antioxidant mechanism of blueberry, together with the adhesive features of clingfish, we present novel wet adhesive proanthocyanidins (pAc) MNs with desirable functionality for ocular wound healing (Fig. 1). In nature, various plants, such as blueberries [18,19], grapes [20], *Solanum tuberosum* [21], and strawberries [22], exhibit antioxidant properties due to the existence of pAc. Recently, pAc has been demonstrated with anti-inflammatory, antioxidant, antibacterial, and anti-allergic abilities, which has attracted broad interest in healthcare and biomedical field [23–26]. Besides, pAc facilitates the production of rhodopsin in retinal cells, thus preventing serious myopia and retinal detachments, and improving visions [27]. Additionally, many natural creatures exhibit exceptional adhesive abilities, attributed to the molecular attraction and/or surface microstructure [28–31]. As a typical instance, clingfish can adhere strongly to almost any substrate underwater through their suctions facilitated by hexagonal microstructure and chemical interactions between their adhesive proteins [32–34]. The hexagonal structures are interconnected by small grooves that expedite the drainage of interfacial water during adsorption, thus allowing for rapid adhesion between the 2 interfaces. Therefore, we conceive that integrating these bioinspired properties into the MNs is expected to develop a new system with both adhesive and therapeutic abilities for treating ophthalmic diseases.

Herein, by adopting the adhesive mechanism of clingfish and the antioxidant property of blueberry, we present the desired multifunctional MNs (MF-MNs) for ocular wound healing. The MNs are constructed using polyacrylic acid, acrylate *N*-hydroxysuccinimide, and gelatin-based hydrogels [PAAc-NHS/gelatin (PNH)] as the flexible base, featuring hexagonal structures. When in contact with wet tissues, PAAc can absorb the liquid at the interface, while excess liquid is drained through the small grooves between hexagonal structures. Simultaneously, NHS can easily crosslink with amino groups in tissues to achieve strong adhesion. Besides, the MN tips, made from gelatin loaded with pAc, impart the system antioxidant properties that effectively scavenge ROS. The developed MNs not only demonstrated great adhesion and desirable antioxidant properties when employed in the wet ocular tissue but also behaved effectively in terms of sustained drug release and treatment in the rat ocular alkali burn model. These results indicated that our MN system can overcome the restrictions of conventional approaches, which often struggle to perform effectively on wet tissues, thus providing a promising perspective for transdermal drug delivery anywhere in the body.

## Results and Discussion

**Fig. 2.**
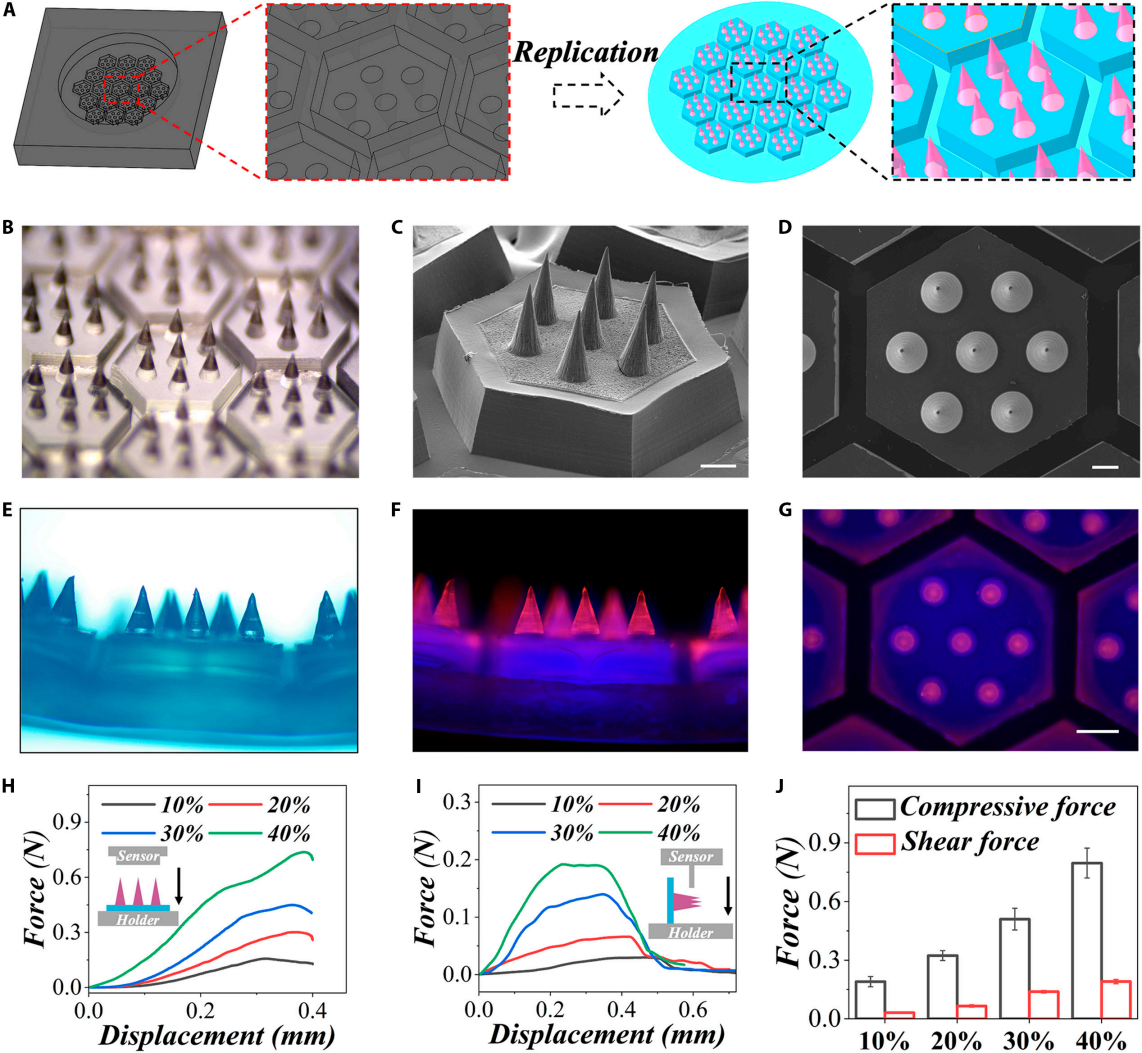
Preparation and characterization of the MF-MNs. (A) The scheme shows the fabrication of the MNs. (B to D) Optical and scanning electron microscopy (SEM) images of the MNs. (E to G) Brightfield and fluorescent images showing the structure of the MNs. Compressive curves (H), shearing curves (I), and corresponding maximum forces (J) of MNs with varying gelatin concentrations. Scale bars, 200 μm (C and D) and 400 μm (E to G).

Generally, the MF-MNs were constructed through the replication of a customized negative mold (Fig. 2A). The mold featured a precise arrangement of hexagons, each containing 7 conical cavities. Gelatin (from porcine skin, with the gel strength of 300 g of Bloom) was used in the whole system since it is a natural biopolymer material obtained by moderate hydrolysis and thermal denaturation of collagen, which has an amino acid sequence similar to that of collagen. After adding gelatin solution into the mold as the MN tip and PNH pregel solution as the MN base, MNs with desirable structures and adhesive properties can be prepared through the photocrosslinking process under ultraviolet (UV) light, as shown in Fig. 2B. The fabricated MNs nicely replicated the architecture of the mold, displaying ordered hexagons and conical needles (Fig. 2C and D). Besides, the structure can also be visualized distinctly in the brightfield and fluorescent images (Fig. 2E to G). Remarkably, such structures could closely resemble the suctions on the clingfish, which also feature hexagonal microstructures. To realize transdermal drug delivery, the MNs require sufficient mechanical properties for penetrating tissues; therefore, the compressive and shearing forces of the MF-MNs were determined using a mechanical testing machine (Fig. 2H to J). To explore the impact of gelatin concentration on these forces, MF-MNs were prepared with tip concentrations of 10%, 20%, 30%, and 40% gelatin. The results indicated that the higher concentration led to increased compression/shearing force. Besides, the MF-MNs with 30% concentration can attain ~0.4-N compressive force, which was sufficient for the penetration of ocular surface [35,36].

**Fig. 3.**
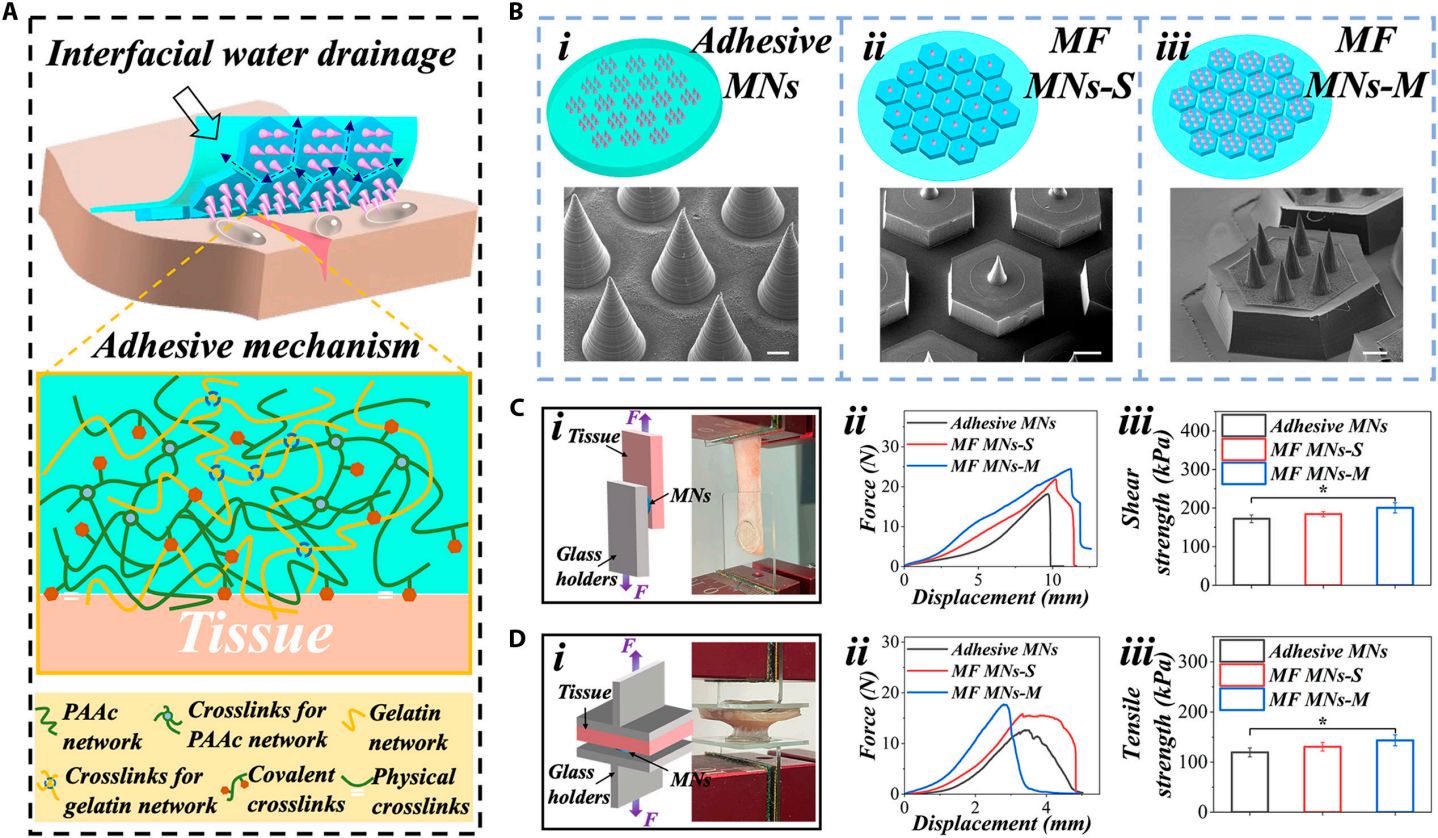
Determination of adhesive property. (A) Schematic illustration showing the adhesive mechanism of the MF-MNs. (B) Scheme and SEM images of 3 various MNs: (i) adhesive MNs, (ii) MF MNs-S, and (iii) MF MNs-M. Determination of adhesive abilities of different MNs on the tissues based on (C) lap shear test and (D) tensile test, respectively. (i) Scheme and optical images exhibiting the adhesive type and process of MNs on the tissue; (ii) curves of force as a function of displacement; (iii) maximum shearing and tensile strengths. Scale bars, 100 μm (B, i), 500 μm (B, ii), and 200 μm (B, iii).

As shown in Fig. 3A, after the MF-MNs were inserted into the wet tissue, the carboxylic acid group in the PNH could quickly absorb the interfacial water between MNs and tissue. The hexagonal structures, interconnected by small grooves, further facilitated the drainage of excess water during this process. Subsequently, the NHS group in the PNH could rapidly crosslink with the amino group in the tissue. Besides, the carboxylic acid group in the PNH could form electrostatic interactions and hydrogen bondings with the tissue [37,38]. These combined effects of interfacial water absorption and removal, along with the chemical and physical interactions, enabled the MF-MNs to adhere strongly to various types of wet tissues. We first measured the tissue adhesive ability of the MF-MNs using the porcine skin, which was brought from the local food market and freshly prepared. A scalpel was used to scrape off the surface oil, and a tweezer was used to pull out the sweaty hairs on the epidermis before use. The final porcine skin used consists of the epidermis, dermis, and part of the subcutaneous fat. From Fig. S1, the MF-MNs can maintain tight adhesion to the skin under various conditions, including suspending, bending, stretching, and submersion underwater. Besides, weights were used to further exhibit the adhesive ability and bearing force of the MNs. After the MNs were inserted into the porcine skin, weights were attached to the opposite side of the MNs. The results indicated that the MF-MNs can easily support loads of over 260 g, and over 20 g underwater (Fig. S2).

The designed MNs demonstrated superior adhesive properties in a variety of situations in comparison to their counterparts. For the evaluation of this, 3 various MNs were fabricated for lap shear and tensile tests. They were MNs with PNH hydrogels as the base but without hexagonal structures (adhesive MNs), MNs with PNH hydrogels as base, and 1 tip in each hexagonal structure [multifunctional MNs-single (MF MNs-S)], and MNs with PNH hydrogel as the base, and 7 tips in each hexagonal structure [multifunctional MNs-multi (MF MNs-M)] (Fig. 3B). We first determined and recorded the variation of shearing force with increasing displacement (Fig. 3C). The results demonstrated a sequential increase in shearing strength from adhesive MNs to MF MNs-S, and finally to MF MNs-M. For the tensile test, MF MNs-M exhibited a maximum tensile strength of 130 ± 1.9 kPa, which was higher than that of MF MNs-S (120.2 ± 2.3 kPa), and significantly higher than that of adhesive MNs (110 ± 3.5 kPa) (Fig. 3D). Therefore, with the integration of PNH hydrogels and hexagonal structures as the base, the resultant bioinspired MF-MNs could not only inherit their merits but also eliminate their disadvantages, providing tightly adhesive abilities and a wide range of application conditions.

To impart MNs with antioxidant properties, pAc was incorporated in MN tips. First, the biocompatibility of pAc was evaluated. Human keratocytes (HKs) and human corneal epithelial cells (HCECs) were cultured with varying concentrations of pAc (0 to 100 μg/ml). Cellular viability and Live/Dead assay were carried out after 1, 2, and 3 d of culture. From Figs. S3 to S5, HKs and HCECs maintained high viability during the culture time, and the presence of pAc could promote the proliferation of the HKs. Because of the presence of phenolic hydroxyl structures in pAc, it could readily liberate protons. The catechol structure at the 3′, 4′ positions on the B ring of pAc forms a stable conjugated semiquinone or o-quinone structure with ROS through 2 consecutive single-electron transfer reactions, evenly distributing the electron cloud and reducing intramolecular energy. The phenolic hydroxyl group at the 5-position of the A ring can easily oxidize and release H+, which has a strong ability to scavenge ROS. Additionally, the phenolic hydroxyl groups at the 3, 5, and 7 positions further interact with ROS to form a pseudo-semiquinone structure, enhancing stability through keto-enol tautomerism. Therefore, pAc has been widely used as an antioxidant for scavenging free radicals. Therefore, pAc has been broadly used for scavenging free radicals as an antioxidant [26,39,40].

**Fig. 4.**
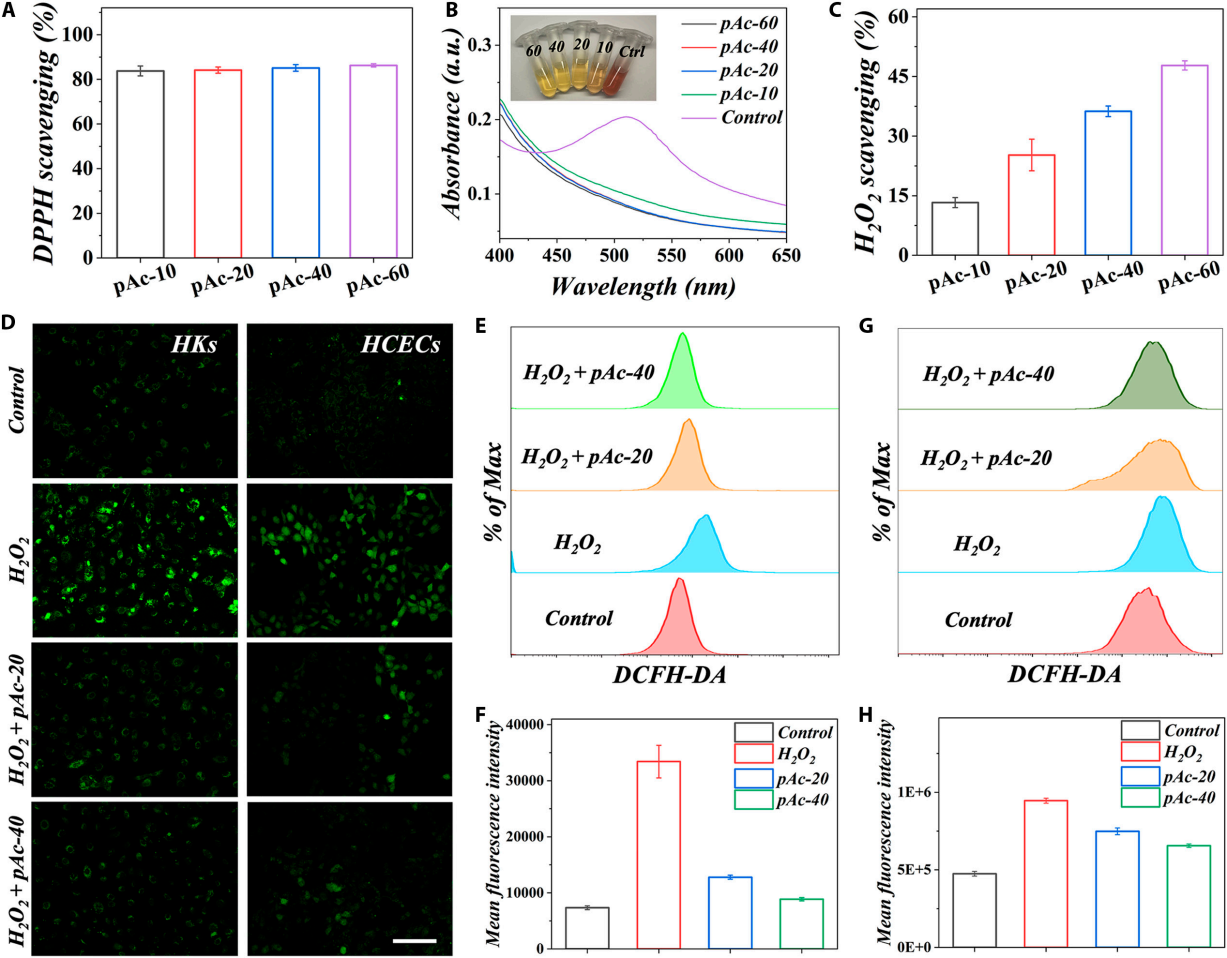
Scavenging ROS with pAc in vitro. (A) Histogram of DPPH scavenging ability. (B) UV-visible absorption spectrum of DPPH treated by different concentrations of pAc. (C) H_2_O_2_ scavenging activity. (D) DCFH-DA staining of HKs and HCECs after being treated with H_2_O_2_ and different concentrations of pAc. (E) DCFH-DA level of HKs after being treated with H_2_O_2_ and different concentrations of pAc. (F) Histogram showing the DCFH-DA mean fluorescent intensity of HKs after treatment in (E). (G) DCFH-DA level of HCECs after being treated with H_2_O_2_ and different concentrations of pAc. (H) Histogram showing the DCFH-DA mean fluorescent intensity of HCECs after treatment in (G). Scale bar, 100 μm (D).

We further determined the antioxidant ability of pAc. It is evident that pAc could substantially improve the scavenging efficiency of 1,1-diphenyl-2-picryl hydrazyl (DPPH) radicals with a corresponding reduction of the characteristic absorption peak at 517 nm in the UV-visible spectrum, as shown in Fig. 4A and B. In addition, pAc also endowed the MNs with the ability to decompose H_2_O_2_ (Fig. 4C). Furthermore, we investigated the cytoprotective ability of pAc to inhibit ROS-induced injury in vitro using HKs and HCECs. First, the green fluorescence probe 2′,7′-dichlorofluorescein diacetate (DCFH-DA) was used to label the ROS in the cells. From Fig. 4D, the ROS level in HKs and HCECs elevated remarkably under the stimulation of 250 μM H_2_O_2_. However, the presence of pAc could decrease the influence of H_2_O_2_ on the cells. In addition, flow cytometry was performed to further evaluate the antioxidant ability of pAc. As shown in Fig. 4E to H, the fluorescent intensity decreased with the addition of pAc, and a higher concentration of pAc resulted in a greater reduction in the intensity. These results collectively demonstrate that pAc possessed strong antioxidant properties.

Since MF-MNs showed high cell survival and excellent antioxidant properties in vitro, they were further employed in a rat model of alkali burns. First, MF-MNs were inserted into the normal corneas of rats, and the resulting corneal structure variation was determined by performing the hematoxylin-eosin (HE) staining. As shown in Fig. S6A, the epithelium of the cornea was readily penetrated by the MNs. In addition, the immunofluorescent staining of the apoptosis marker terminal deoxynucleotidyl transferase–mediated deoxyuridine triphosphate nick end labeling (TUNEL) was performed to evaluate the influence of the penetration process on the corneal cells. Figure S6B indicated that the insertion of MNs had no influence on the corneal cells. We further determined the biocompatibility of MNs in vivo by embedding them into subcutaneous tissue. HE staining was performed to observe the main tissues of the rats, and there was no apparent variation between the normal tissue and the embedded one (Fig. S7).

After confirming biocompatibility in vivo, we verified the therapeutic potential of the MF-MNs in an alkali burn model induced by sodium hydroxide (NaOH) in Sprague–Dawley (SD) rats. Adhesive MNs without pAc and MF-MNs were subsequently inserted into the burned cornea. In the control group, the cornea was untreated after burns. These rats received no additional treatment afterward. Ocular variations in the rats were evaluated by a clinician and recorded with a surgical microscope.

**Fig. 5.**
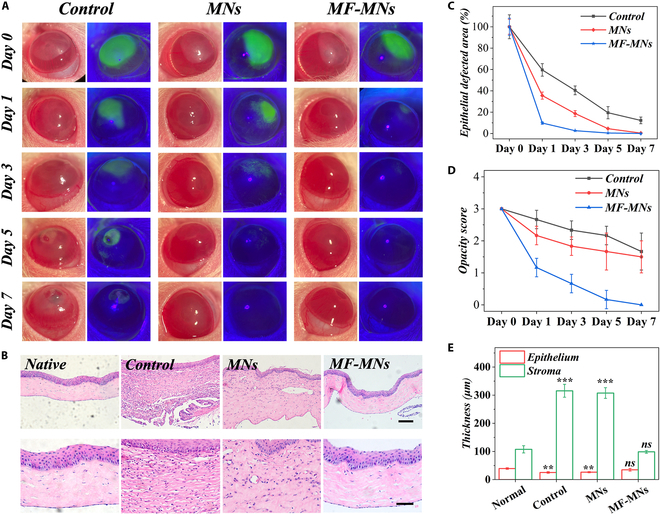
In vivo evaluation of the MF-MNs. (A) Representative photographs and fluorescein staining images of burned corneas under different treatments. (B) Histogram of epithelial defected area. (C) Opacity score. (D) HE staining images of native corneas and burned corneas with different treatments on day 7. (E) Thickness histogram of epithelium and stroma. Scale bars, 100 μm (D) and 50 μm (enlarged images).

On day 1, fluorescein staining revealed obvious epithelial defects in both untreated and MN-treated eyes (Fig. 5A). These defects persisted in the control group, with a 12% defected area still present on day 7. However, there was no staining in the MF-MN group on day 5, indicating the repair of the epithelium (Fig. 5C). Besides, the opacity score was recorded (Fig. 5D). The opacity in MF-MN-treated eyes markedly decreased on day 1, with gradual clarity restored over the 7-d treatment period.

Corneas were collected after 7 d of treatment, and HE staining was performed to evaluate the corneal epithelium and stroma. From Fig. 5B and E, the corneas in the control and MN-treated groups exhibited obviously reduced thickness of epithelium and increased thickness of the stromal layer compared to native cornea, indicating that they were still in the state of edema. However, the corneas in the MF-MN-treated groups showed similar thickness to that of native cornea. In addition, prominent inflammatory cells were present in the control and MN-treated groups, whereas few could be observed in the MF-MN-treated group.

**Fig. 6.**
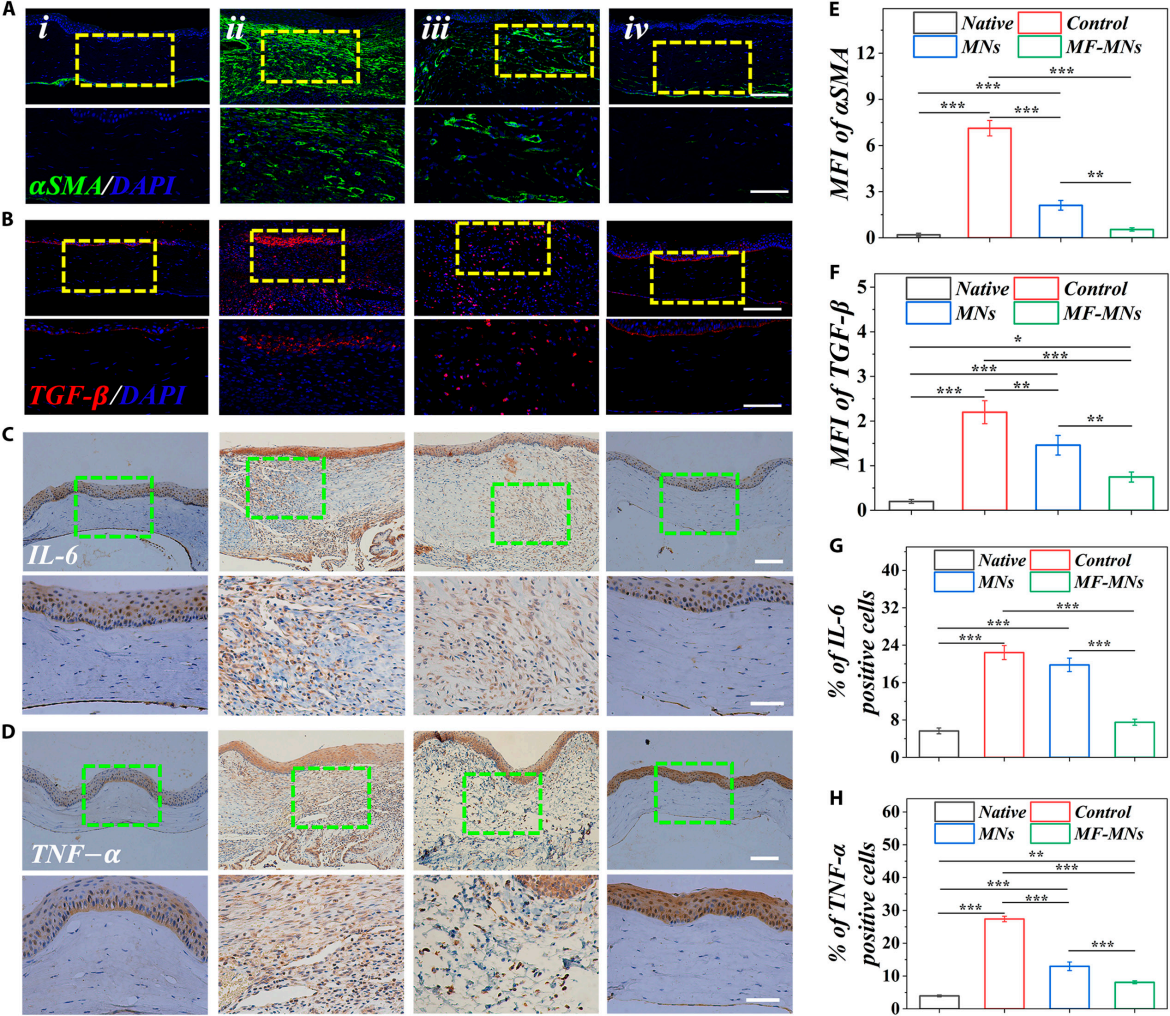
MF-MNs reduce ocular tissue inflammation after alkali burn. Representative immunofluorescent (A) αSMA and (B) TGF-β and immunohistochemical (C) IL-6 and (D) TNF-α staining images of burned corneas under different treatments. (i) Native cornea. (ii) Group without treatment. (iii) Group treated with MNs. (iv) Group treated with MF-MNs. Quantification of (E) αSMA, (F) TGF-β, (G) IL-6, and (H) TNF-α percentage. Scale bars, 100 μm (A to D) and 50 μm (enlarged images).

To further investigate the molecular mechanisms underlying corneal healing after treatment with MF-MNs, we performed the immunofluorescent staining of α-smooth muscle actin (αSMA), specifically expressed in the myofibroblast, and transforming growth factor-β (TGF-β), the inductive cytokine of myofibroblast, which highlighted fibrosis of tissues and formation of scars [1,2]. In the control and MN-treated groups, strong αSMA and TGF-β staining were observed in the whole corneas. In contrast, sparse αSMA and TGF-β staining were present in the MF-MN-treated corneas (Fig. 6A, B, E, and F). Besides, alkali burns led to significant infiltration of CD45^+^ leukocytes in the corneas, whereas this was markedly reduced in the MF-MN-treated group (Fig. S8A and C). Furthermore, it was observed that alkali burns could increase the expression of inflammatory cytokines interleukin-6 (IL-6), tumor necrosis factor-α (TNF-α), and inflammatory mediator matrix metalloprotein 9 (MMP9), respectively (Fig. 6C, D, G, and H and Fig. S8B and D), in the cornea after burn. MF-MN treatment could dramatically reduce the levels of these factors. All the results indicate that our designed MF-MNs have great potential for the treatment of ocular wound healing.

## Conclusion

In summary, by integrating the adhesive mechanism of clingfish and the antioxidant property of blueberry, we constructed desirable MF-MNs for ocular wound healing. The MNs feature PNH hydrogels as a base with hexagonal structures. When contacting with wet tissue, PAAc can absorb liquid in the interface, while excess liquid is efficiently drained through the small grooves between hexagonal structures. Simultaneously, NHS can easily crosslink with amino groups in tissues to achieve strong adhesion. Besides, gelatin was selected as the material for MN tips, which were loaded with pAc to endow the MNs with antioxidant properties to scavenge ROS. It was verified that the developed MNs not only exhibited great adhesion and desirable antioxidant properties when employed in the wet ocular tissue but also behaved effectively in terms of sustained drug release and treatment in the rat ocular alkali burn model. These results suggest that bioinspired MNs with multifunctions can overcome the restrictions of conventional approaches and become promising candidates for versatile wound healing systems.

## Materials and Methods

Details about the methods used in this research are available in the Supplementary Materials.

## Data Availability

The data that support the findings of this study are available from the corresponding author upon reasonable request.

## Supplementary Materials

Supplementary 1 Experimental Section Figs. S1 to S8
