# Supplementary material for: Bioinspired Wet Adhesive Proanthocyanidins Microneedles for Ocular Wound Healing
Source: Research (Wash D C). 2024 Sep 24;7:0485. doi: 10.34133/research.0485 (PMC11420907; doi:10.34133/research.0485)
Supplement: Supplementary 1 — Experimental Section Figs. S1 to S8 [file research.0485.f1.docx]

**Supplementary**

**Bioinspired wet adhesive proanthocyanidins microneedles for ocular wound healing**

Bin Kong^1,2,3^, Rui Liu^2^, Tiantian Kong^1,3,^*, Yuanjin Zhao^2,^*

1 School of Biomedical Engineering, Shenzhen University Medical School, Shenzhen University, Shenzhen 518060, China

2 Department of Rheumatology and Immunology, Nanjing Drum Tower Hospital, School of Biological Science and Medical Engineering, Southeast University, Nanjing 210096, China

3 Department of Urology, Inst Translat Med, The First Affiliated Hospital of Shenzhen University, Shenzhen Second People's Hospital, Shenzhen, Guangdong 518000, China

Email: [yjzhao@seu.edu.cn](mailto:yjzhao@seu.edu.cn) (Y.J.Z.); ttkong@szu.edu.cn (T.T.K.)

**Experimental Section**

*Materials*

aa-PEG-NHS was provided by Ponsure Biological Corporation (China). pAc and NaOH were bought from Aladdin (China). ﻿1,1-diphenyl-2-picryl hydrazyl (DPPH) assay kit was obtained from Nanjing Jiancheng Bioengineering Institute (China). ﻿ROS assay kit 2’,7’-dichlorofluorescein diacetate (DCFH-DA), and HE staining kit were bought from Beyotime (China). Sodium fluorescein was obtained from Tianjin Yinuoxinkang Medical Device Tech Co., Ltd (China). All other chemical materials not mentioned were purchased from Sigma-Aldrich (USA). HCECs and HKs were provided by the Chinese Academy of Sciences. The cell culture-related materials, including Dulbecco’s modified Eagle’s medium (DMEM), Fetal Bovine Serum (FBS), Penicillin-Streptomycin (PS), Phosphate Buffer Saline (PBS), and trypsin were bought from Gibco (USA). The cell Live-Dead assay kit and CCK-8 were purchased from Thermofisher (USA). The primary antibodies of αSMA, TGF-β, CD45, MMP9, TNF-$\alpha$, and IL6 were bought from Proteintech (USA). The secondary antibodies of Alexa Fluor 488 goat anti-rabbit IgG H&L, Alexa Fluor 594 goat anti-mouse IgG H&L, and HRP-conjugated goat anti-rabbit IgG H&L were bought from Abcam (USA). ﻿The 6 weeks male Sprague–Dawley (SD) rat was bought from the Model Animal Research Center of Nanjing University.

*Fabrication and characterization of the MNs*

﻿To fabricate MF MNs, gelatin solution with pAc was first filled into the tip of a custom-built polydimethylsiloxane (PDMS) negative mold under a vacuum, and the residual solution was removed. Then, the PNH pregel solution prepared by 30% aa, 10% gelatin, 1% aa-PEG-NHS, 0.1% crosslinker BIS, and 1% photoinitiator HMPP (w/w) was filled into the hexagonal caves of the mold and then exposed to the UV light (365 nm, 3W) for 20 min to form the wet adhesive PNH. Finally, the filled template was dried at room temperature, and the resultant MNs (200 μm in diameter and 400 μm in length) with an ordered array of hexagon chambers were gently peeled out of the negative mold. The MNs were stored under dry environment at room temperature for further use. The optical images were obtained using a stereomicroscope. The fluorescent images were taken by a biological microscope (Leica, Germany). The ﻿microscopic morphology of the needle was captured by an SEM (ZEISS, Germany). ﻿

*Mechanical strength of the MNs*

﻿The mechanical strength of the MF MNs with the gelatin concentration of 10%, 20%, 30%, and 40% was determined by a mechanical testing machine (﻿INSTRON, Germany). For the measurement of compressive force, the MNs were placed on the immobile clamp, with the orientation of the tips vertically to the movable clamp. For the measurement of shear force, MNs were fixed on a self-designed clamp, which can maintain the orientation of the tips horizontally to the movable clamp, which was attached with a sharp blade. The loading speed was set as 0.2 mm/min.

*Adhesive test of the MNs*

The shear strength and tensile strength of the adhesive MNs, MF MNs-S, and MF MNs-M on the porcine skin were measured through the standard lap-shear test (ASTM F2255) and tensile test (ASTM F2258), respectively, by using the mechanical testing machine. Notably, a small amount of PBS was added to the porcine skin to trigger the adhesion before pressing the MNs on the tissues for 10 s. For the measurement of shear strength, the rectangular porcine skin with a width and length of 10 and 40 mm was prepared. After adhering to the porcine skin, the other side of the MNs was fixed to a glass slide. The shear strength was calculated by the following formula: shear strength=Fmax/adhesion area, where Fmax is the maximum force. For the measurement of tensile strength, the square porcine skin with a width and length of 20 mm was prepared. After adhering to the porcine skin, the other side of the MNs was fixed to a glass slide. The tensile strength was calculated by the following formula: tensile strength=Fmax/adhesion area, where Fmax is the maximum force. ﻿

*In vitro biocompatibility*

To evaluate the biocompatibility of pAc-loaded MNs, the MNs with the concentrations of pAc 0, 10, 20, 40, 60, 80, and 100 μg/mL were used to culture HKs and HCECs for 1, 2, and 3 days, respectively. At each time point, a Live/Dead assay was performed by staining the cells with the mixture of ethidium homodimer and calcein AM solution, followed by the observation by a fluorescent microscope (Leica, Germany). The Cell Counting Kit-8 was utilized to determine cell viability. In brief, the cells were incubated in the mixture of CCK-8 solution and culture media in the dark for 2 h at 37$℃$, followed by the measurement of the OD at 450 nm by using a plate reader. ﻿

In addition, MF MNs were inserted into the eyeballs of the SD rats to determine the penetrating ability and biocompatibility of the MNs to the native tissue. After inserting for 4 h, the eyeball was harvested from the rats, and cornea was obtained. The cornea was then fixed into 4% paraformaldehyde and dehydrated into 30% sucrose, followed by a freezing process into 10-μm sections using a freezing microtome (Leica, Germany). The sections were performed with HE staining and tunel/DAPI staining, respectively.

*In vitro antioxidant experiments*

*﻿*The DPPH free-radical scavenging ability of pAc was detected using the DPPH assay kit. In brief, the single electron in DPPH reacts with an alcohol solution to appear purple in color, which displays an absorbance peak at 517 nm. When free radical scavengers are present, their pairing with the single electron in DPPH causes the color to be lighter. Various concentrations of pAc (10–60 μg/mL) were reacted with 80% methanol at room temperature for 30 min, respectively. After the reaction, the eliminating capacity was calculated according to the manufacturer’s instructions. The H_2_O_2_ scavenging ability of pAc was detected using ﻿a hydrogen peroxide assay kit. In brief, pAc was incubated in the 2 mM H_2_O_2_ solution for 2 h at 37$℃$. Then the absorbance of treated H_2_O_2_ solutions was detected at 405 nm by a plate reader. At last, the concentration of H_2_O_2_ was calculated according to the manufacturer’s instructions. To further investigate the intracellular antioxidant capabilities of the pAc, DCFH-DA, a green fluorescent probe for ROS, was employed to investigate intracellular ROS levels. HKs and HCECs were seeded in confocal dishes and grown overnight. After removing the cell culture medium, the adherent cells were exposed to the identical volume of H_2_O_2_ or concentrations of pAc (20 and 40 μg/mL) for 6 hours. The fluorescent probe DCFH-DA was then introduced. Afterward, fluorescence microscopy was used for cell imaging. For quantitative assessments, cells in 6-well plates received identical treatment as described earlier, aimed at detecting intracellular ROS. Following a 20-minute DCFH-DA exposure period, these cells underwent trypsinization and were then washed three times with PBS. The fluorescence intensity in the cells was later quantified through flow cytometry.

*In vivo antioxidant evaluation*

﻿ All the animal procedures were performed in compliance with the guidelines of the Institutional Animal Care and Use Committee of the Department of Nanjing University. SD rats were anesthetized by isoflurane, and one drop of lidocaine hydrochloride was topically applied to the right eye for 1 min. A sterile 3.5-mm-diameter filter paper was soaked in 1 mol/L sodium hydroxide solution for 10 s. Excess sodium hydroxide was removed by a paper towel. The filter paper was then applied onto the central cornea for 20 s, followed by irrigating the ocular surface with PBS until the pH level of the ocular surface returned to 7. The rats were randomly divided into 3 groups of 6 rats each. MNs and MF MNs were applied to the burned cornea respectively, and the cornea without treatment was used as a control group. After the rat woke up, antibiotic eye ointment was applied, topically to prevent infection. No additional ocular treatment was given afterward. Immediately, 1, 3, 5, and 7 days after the operation, the MNs were removed and the eyes were then examined using a surgical microscope. Besides, corneal fluorescein staining was used to evaluate corneal epithelial damage using the surgical microscope ﻿under blue light. The corneal epithelial defected area was determined by measuring the stained areas using the software of Image J. In addition, optical opacity was scored based on the clinical standard.

On day 7, the corneas were separated for HE and histochemical staining. All tissues obtained were fixed in 4% formaldehyde and then dehydrated in a series of gradient ethanol before vitrification by dimethylbenzene. The treated tissues were embedded in paraffin and sliced into slides with a thickness of 5 μm ﻿by a microtome. For HE staining, the slices were deparaffinized, rehydrated, and then immersed into the specific staining solutions according to the manufacturer’s manual, respectively. For immunohistochemistry analysis, the slices were deparaffinized, rehydrated, and then incubated with primary antibodies (IL-6 and TNF-$\alpha$) and secondary antibodies, followed by staining with DAB and hematoxylin. For immunofluorescence staining, the slides were deparaffinized, rehydrated, and then incubated with primary antibodies ($\alpha$SMA, TGF$\beta$, MMP9, and CD45) and secondary antibodies, followed by the mounting with DAPI solution. The slides were observed under fluorescent microscopy (Leica, Germany).

*Statistical analysis*

All the results are presented as the means ± standard deviation. Statistical analysis was carried out using T-tests or one-way ANOVA followed by a post-hoc test to determine the degree of significance. *P< 0.05, **P < 0.01; ***P < 0.001.

***Figures***


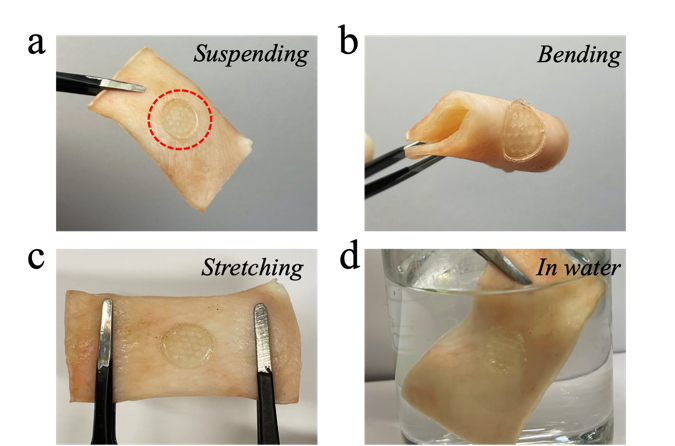


**Figure S1.** Representative adhesive images of MF-MNs on the porcine skin when they were (a) suspending, (b) bending, (c) stretching, and (d) in water.


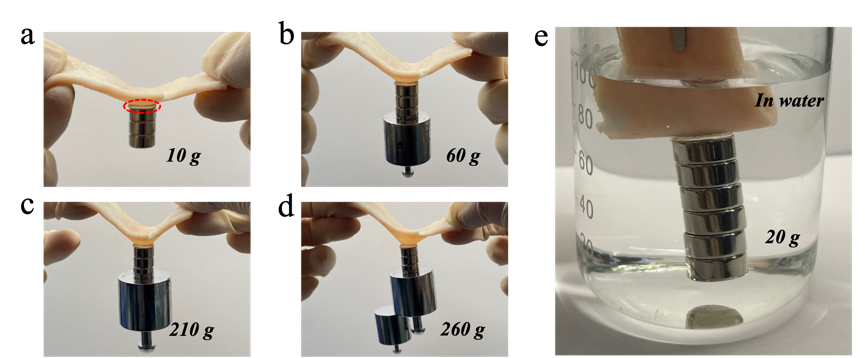


**Figure S2.** The MF-MNs adhered to the porcine skin when the weights were (a) 10 g, (b) 60 g, (c) 210 g, (d) 260 g, and (e) 20 g in water.


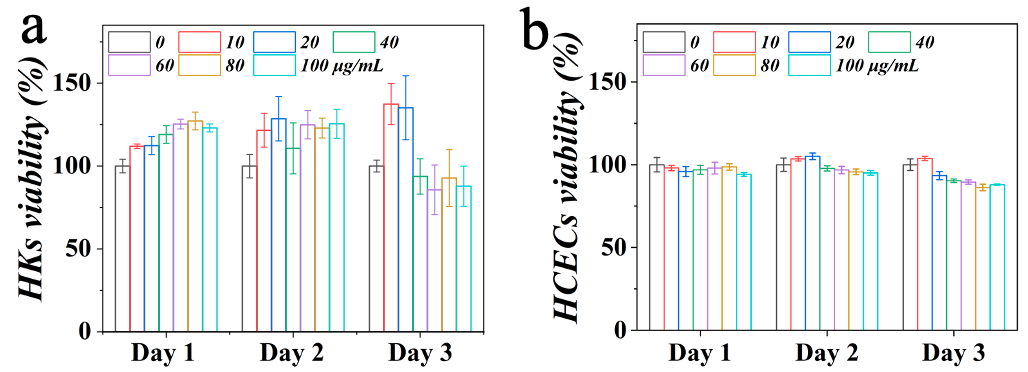


**Figure S3.** The cell viability of (a) HKs and (b) HCECs with different concentrations of pAc after culturing for 1, 2, and 3 days.


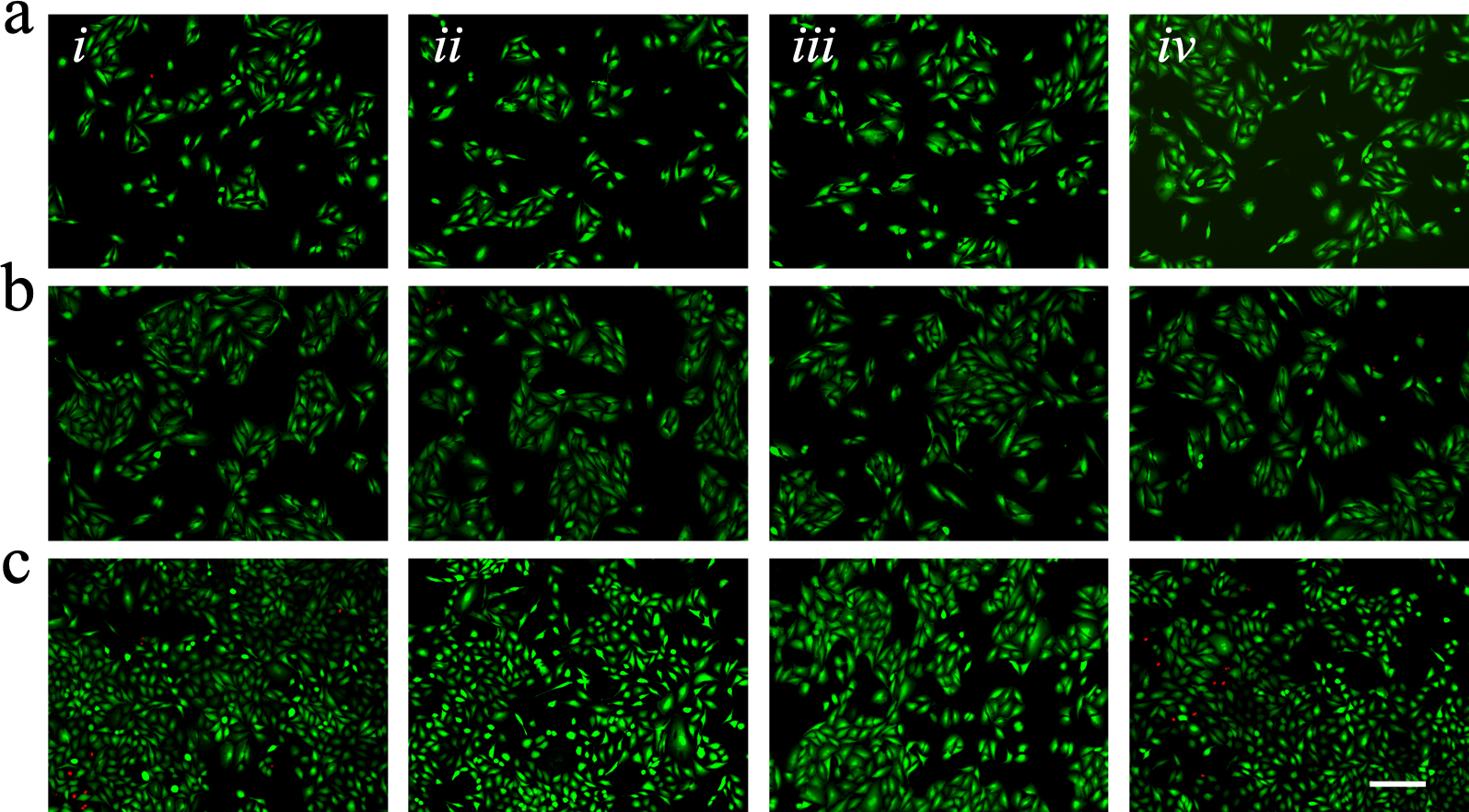


**Figure S4.** The Live/Dead staining images of HKs after culturing with different concentrations of pAc. i) 0, ii) 10, iii) 20, and iv) 40 μg/mL. The scale bar is 200 μm.


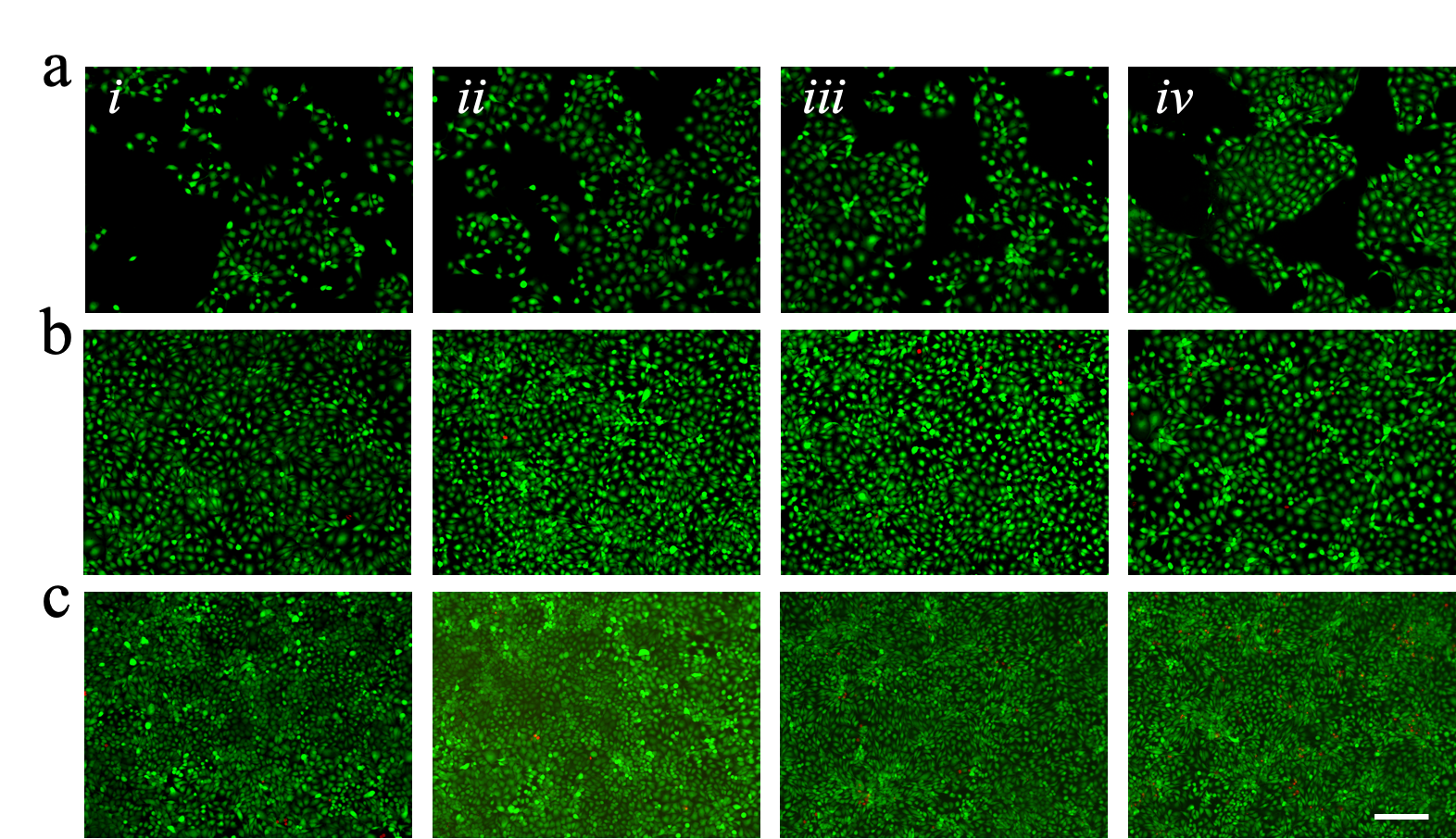


**Figure S5.** The Live/Dead staining images of HCECs after culturing with different concentrations of pAc. i) 0, ii) 10, iii) 20, and iv) 40 μg/mL. The scale bar is 200 μm.


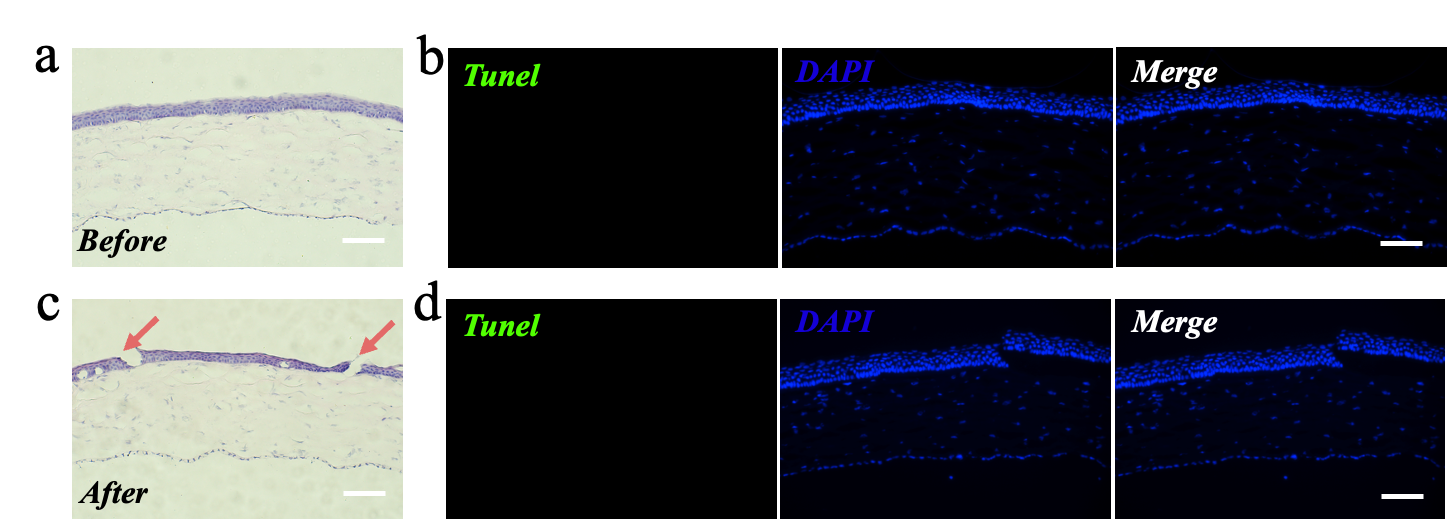


**Figure S6.** Representative (a, b) HE staining and (c, d) tunel/DAPI staining images of rat cornea before and after MNs insertion. The scale bars are 100 μm.


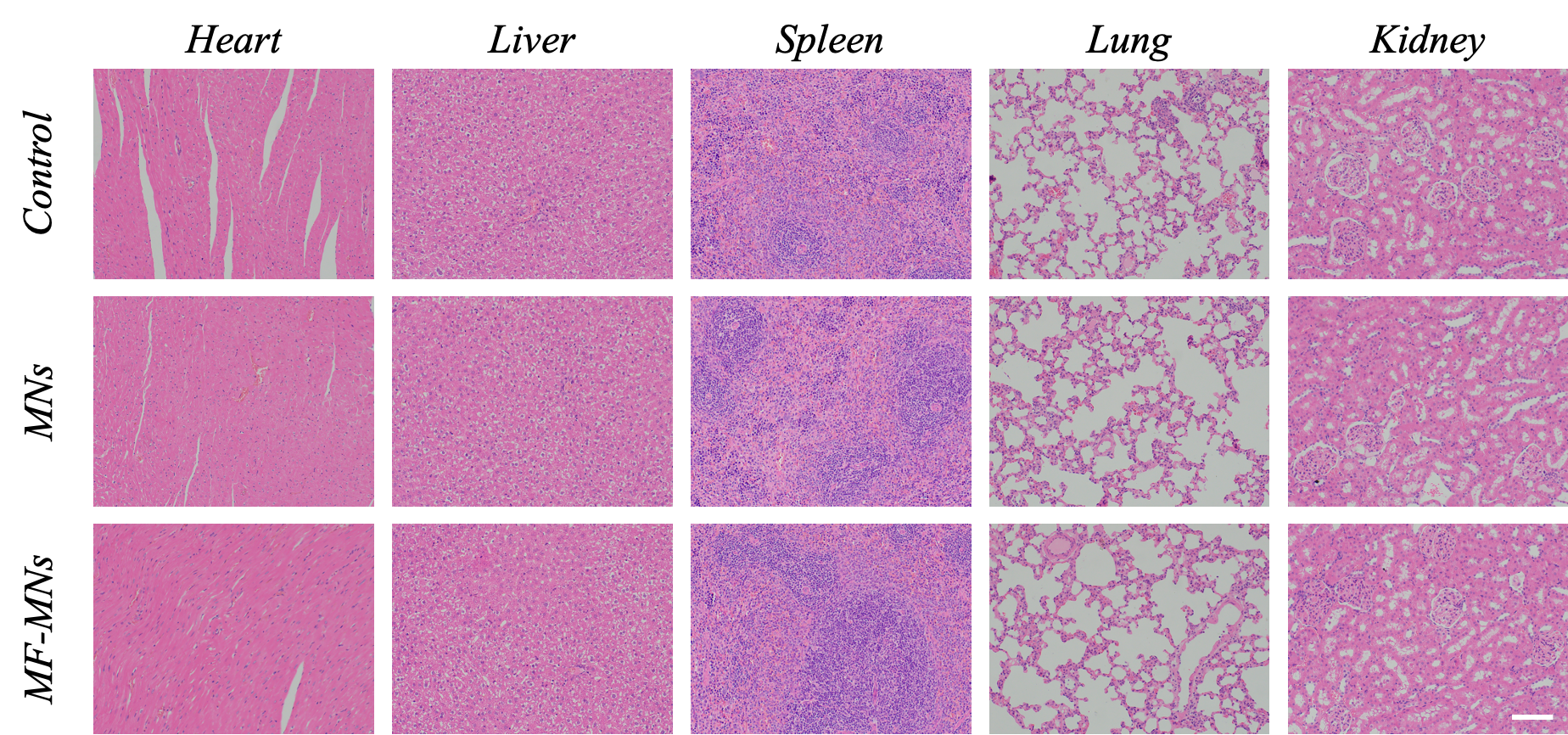


**Figure S7.** Representative HE staining images of heart, liver, spleen, lung, and kidney from normal rats (control), and rats subcutaneously implanted with MNs and MF-MNs for 2 weeks, respectively. The scale bar is 100 μm.


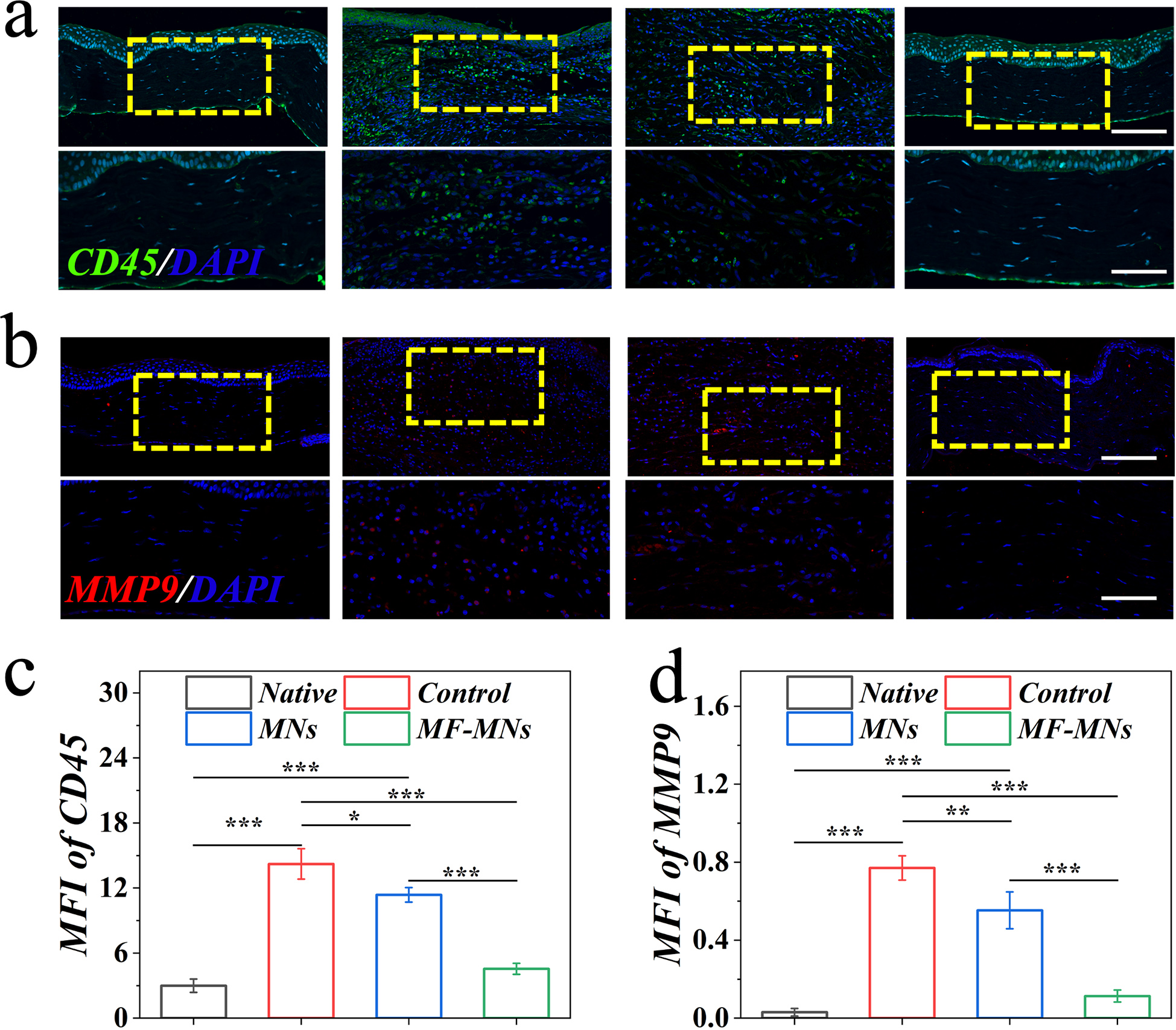


**Figure S8.** ﻿Representative immunofluorescent (a) CD 45 and (b) MMP9 staining images of infected corneas after being treated with iii) MNs and iv) MF-MNs for 7 days. i) was the native cornea, ii) was the group without treatment. Quantification of (c) CD 45 and (b) MMP9 percentage. Scale bars: 100 μm in (a, b), and 50 μm in the enlarged images.
